# All-solid-state high performance asymmetric supercapacitors based on novel MnS nanocrystal and activated carbon materials

**DOI:** 10.1038/srep23289

**Published:** 2016-03-29

**Authors:** Teng Chen, Yongfu Tang, Yuqing Qiao, Zhangyu Liu, Wenfeng Guo, Jianzheng Song, Shichun Mu, Shengxue Yu, Yufeng Zhao, Faming Gao

**Affiliations:** 1 Key Laboratory of Applied Chemistry, College of Environmental and Chemical Engineering, Yanshan University, Qinhuangdao, 066004, China; 2 State Key Laboratory of Advanced Technology for Materials Synthesis and Processing, Wuhan University of Technology, Wuhan, 430070, China

## Abstract

All-solid-state high-performance asymmetric supercapacitors (ASCs) are fabricated using γ-MnS as positive electrode and porous eggplant derived activated carbon (EDAC) as negative electrode with saturated potassium hydroxide agar gel as the solid electrolyte. The laminar wurtzite nanostructure of γ-MnS facilitates the insertion of hydroxyl ions into the interlayer space, and the manganese sulfide nanowire offers electronic transportation channels. The size-uniform porous nanostructure of EDAC provides a continuous electron pathway as well as facilitates short ionic transportation pathways. Due to these special nanostructures of both the MnS and the EDAC, they exhibited a specific capacitance of 573.9 and 396 F g^−1^ at 0.5 A g^−1^, respectively. The optimized MnS//EDAC asymmetric supercapacitor shows a superior performance with specific capacitance of 110.4 F g^−1^ and 89.87% capacitance retention after 5000 cycles, a high energy density of 37.6 Wh kg^−1^ at a power density of 181.2 W kg^−1^ and remains 24.9 Wh kg^−1^ even at 5976 W kg^−1^. Impressively, such two assembled all-solid-state cells in series can light up a red LED indicator for 15 minutes after fully charged. These impressive results make these pollution-free materials promising for practical applications in solid aqueous electrolyte-based ASCs.

Supercapacitor, as an advanced energy storage device possesses multiple desirable properties including high power density, fast charging/discharging capability and excellent cycling stability, which hopefully meet the exponentially growing demand of consumer electronics^1^,^2^. However, up to now, most commercially available supercapacitors with a low energy density (<10 Wh kg^−1^), which has restricted their application as primary power sources to replace batteries^3^,^4^. Note that, the energy density (*E*), which denoted as *E* = *CV*^2^*/2,* can be improved by increasing the specific capacitance (*C*) of electrode materials and/or extending the operating potential window (*V*). Currently, two strategies are used to extend the operating potential window: using organic electrolytes (up to 4 V) or developing asymmetric supercapacitor (ASCs)^4^. As compared to aqueous electrolyte, organic electrolytes can provide a better electrochemical stability for electrodes, which however usually suffer from limited ionic conductivity, poor safety and toxicity^5^,^6^. Therefore, ASC design in aqueous electrolytes is an efficient approach to extend the operating potential window and provide effective energy density. These asymmetric supercapacitors are usually composed of a battery-type Faradaic electrode (as the energy source) and a capacitor-type electrode (as the power source), which offering the superiorities of both the battery-type material (energy density) and the capacitor-type material (cycle life, electron transfer rate)^7^,^8^. Meanwhile, ASCs can make full use of the different potential windows of the electrode materials, accordingly provide a maximum potential window in the cell system^7^,^9^. Therefore, selecting proper materials for both positive and negative electrodes to assemble a high performance ASCs are critical.

Hitherto, transition metal oxides/sulfides have been extensively investigated as the positive electrode materials due to their high pseudocapacitance^1^,^6^,^10^,^11^. Among them, manganese sulfide (MnS) nanocrystals have drawn increasing attention because of its remarkable predominance such as high theoretical specific capacitance, inexpensive, environmental friendliness, and higher electronic conductivity (as high as 3.2 × 10^3^ S/cm) than their oxides or hydroxides^1^,^5^,^12^. In addition, the laminar nanostructure (especially γ-phase with a wurtzite structure) accelerates the penetration of electrolyte and the intercalation of ions, which promotes its intrinsic electrochemical reactivity for the capacitive behavior vastly (Fig. 1a). For instance, we have successfully synthesized γ-phase MnS nanocrystal by tuning the sulfide ion content with ammonia as the complex agent and precipitator, of which the specific capacitance reached 704.5 F g^−1 ^11, which is much higher than MnO_2_ (310 F g^−1^ at 2 mV s^−1^)^13^, Mn_3_O_4_ (314 F g^−1^ at 2 mV s^−1^)^14^.

It is generally known that carbonaceous materials are widely used as negative electrode materials, because of their high surface area, excellent conductivity and low cost. However, compared to positive electrode materials (such as transition-metal oxides, sulfides *et al.*)^10^,^15^,^16^, they usually present much lower specific capacitance (e.g. actived carbon, carbide derived carbons, graphene, and so on)^1^,^2^,^3^,^17^,^18^, which restricts the overall performances of ASCs largely. The capacitance of carbonaceous material depends on its structure, because of the capacitance comes from charge accumulation at the interface of electrode material and electrolyte. It is therefore necessary for researchers to develop a carbonaceous material with proper structure. Such as Qie *et al.*^19^ synthesized 3D hierarchical porous carbon, resulting in a high specific capacitance of 318.2 F g^−1^ was achieved.

Herein, we developed an all-solid-state high-performance ASC with agar gel as the solid electrolyte by employing novel MnS nanocrystal and porous eggplant derived activated carbon (EDAC) with high specific capacitance as positive and negative electrodes, respectively. The rod-like structure of MnS nanocrystals provide electronic transmission channels (Fig. 1a); meanwhile, the higher BET specific surface area, micropore area and pore volume of the EDAC (Table 1) can promote the reversible adsorption/desorption of electrolyte ions on the surface of the carbonaceous materials, accordingly providing not only an enhanced power/energy density but also a high rate capability^20^. Because of these specific nanostructures, the MnS nanocrystal and the EDAC electrodes showed high specific capacitances of 573.9 and 396 F g^−1^ at 0.5 A g^−1^ in 2.0 M KOH electrolyte with a conventional three electrode system, respectively. In asymmetric supercapacitor, MnS//EDAC shows a superior device performance with specific capacitance of 110.4 F g^−1^ and 89.87% specific capacitance retained after 5000 cycles. Importantly, the device exhibits a high energy density of 37.6 Wh kg^−1^ at a power density of 181.2 W kg^−1^ and remains 24.9 Wh kg^−1^ at 5976 W kg^−1^.

## Results and Discussion

**Figure 1:**
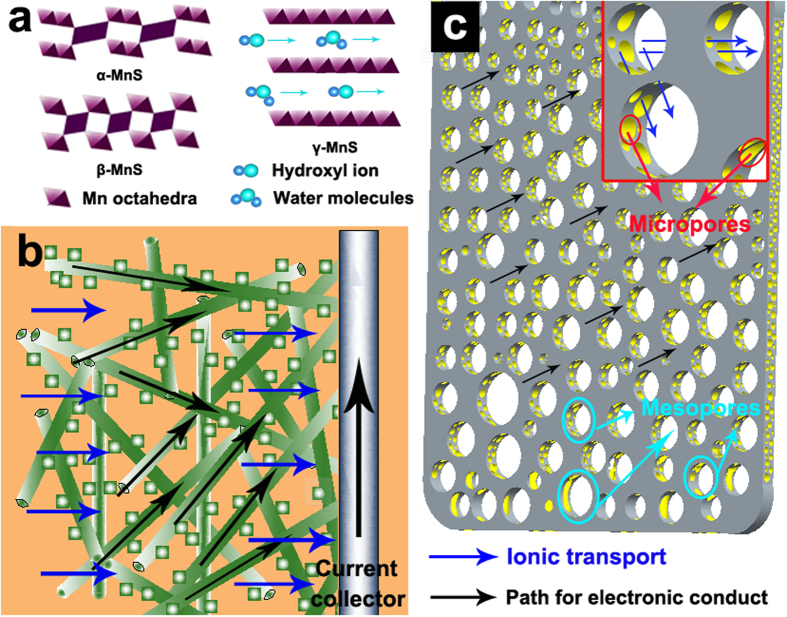
(a) Structures of manganese sulfide: α-MnS (2 × 1tunnel), β-MnS (1 × 1tunnel) and γ-MnS (laminar structure), (b) Suitable structure of γ-MnS nanocrystal and (c) polyporous EDAC for electrochemical reactions in ASCs.

**Figure 2:**
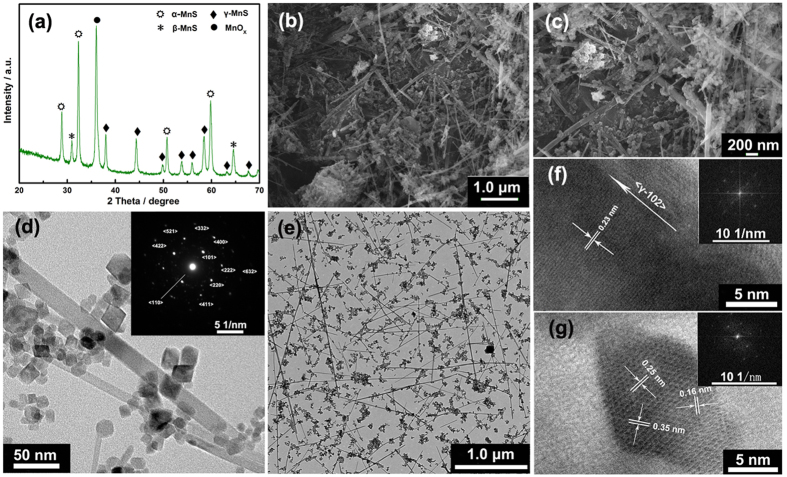
(**a**) XRD pattern of MnS nanocrystal, (**b**) low-magnification, (**c**) high-magnification FESEM images and (**d**) high-magnification (inset is selected area diffraction), (**e**) low-magnification TEM images of MnS nanocrystal, (**f,g**) HRTEM image of MnS nanocrystal (insets is its FFT plots).

**Figure 3:**
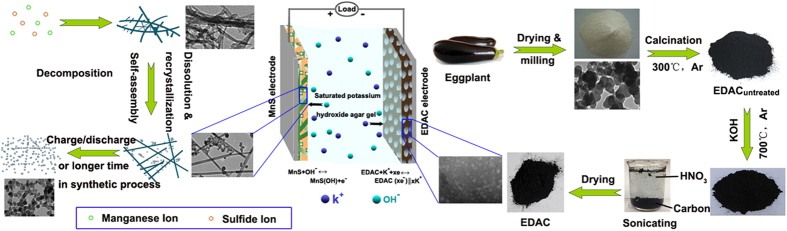


The composition and phase structure of MnS nanocrystal were characterized by the X-ray diffraction (XRD) patterns, as shown in Fig. 2a. Characteristic peaks of MnS showed a highly crystalline character, which were composed mainly of γ- and small amount of α- and β-phases^1^,^11^. As shown in Fig. 1a, manganese sulfides have very diverse structures via sharing corners or edges. Rock salt-type α-MnS (2 × 1) and zinc blende-type β-MnS (1 × 1) composed by edge- and corner-sharing Mn octahedra with tunneled structures, which were unsuitable for the transportation of molecules and ions. Wurtzite-type γ-MnS mainly composed of edge-sharing Mn octahedra, possesses a laminar structure. This structure permits water molecules or hydroxyl anions to easily transfer into/out of the interlayer region, thus more accessing to the total number of Mn ions and exhibiting higher capacitance^11^,^21^,^22^. The γ phase, predominantly caused by the metastable forms (β- and γ-), can be easily precipitated from an aqueous solution in a low-temperature range^23^. Once the temperature or pressure rise to a point or reaction in the reductive condition, the metastable phases will transform irreversibly into stable form. The transformation follows the order: zinc blende (β-)rock salt and wurtzite (α-, γ-)rock salt (α-)^24^. Here, the MnS nanocrystal was obtained at 120 °C in 100 mL Teflon-lined stainless-steel autoclave without reductive agent (Experimental Section in SI). Under this reaction condition, the aqueous solution will evaporate partly, causing the increase of pressure and leading to the transformation accordingly, and then forming MnS nanocrystals contains α-, γ-MnS and little β-MnS. Figure 2b,c show the FESEM images of MnS nanocrystal at different magnifications. It can be seen that, the MnS nanocrystals were mainly composed of nanowires with the lengths of 0.5~3 μm and the diameters of 10~20 nm, with partly nanoparticles (10~20 nm in size) sticking on them. The wire-like MnS nanocrystal (Fig. 1b) provides an electronic transfer channel, which is expected to improve the conductivity and rate capacity of the obtained MnS nanocrystal. Based on this special structure and the similar size of nanowires and nanoparticles, we propose the growth mechanism for MnS nanocrystal, which is shown in Fig. 3. When the thiourea decomposed by heating in alkaline environment, the generated sulfide ions combine with the manganese ions and the MnS precipitation is formed accordingly (Eqs S1~3). With increasing reaction time, the chemical etching will result in dissolution and recrystallization of MnS precipitation, and the wire-like structures are obtained under the action of a orientation force. As the reaction time increased to 5 h or longer (Fig. S1), the MnS nanowires would decompose into nanoparticles. The mechanism of the decomposition can be described as follow: because of the high surface free energy of wire-like MnS, it would combine with the hydroxyl in solution and transform into MnSOH. Due to the larger lattice size of MnSOH than MnS, the decomposition caused by the expansion of the lattice will occur with the increasing of reaction time. The TEM images (Fig. 2d,e) also revealed the wire-like structure of MnS and its excellent dispersibility. In addition, the selected-area electron diffraction (inset of Fig. 2d) indicates the poly-crystalline characteristic of the MnS nanocrystal, which is consistent with the XRD results. Along the direction of the nanowires, the lattice fringe distance is ~0.23 nm in high resolution TEM (HRTEM) image (Fig. 2f), which is corresponding to the (102) planes of γ-MnS. The lattice fringe distances in MnS nanoparticles are 0.16, 0.25, and 0.35 nm, close to that of the γ(004), γ(220), and α(100), respectively. Meanwhile, the FFT plots (insets of Fig. 2f,g) further confirm the poly-crystalline characteristic of MnS nanocrystals.

**Figure 4:**
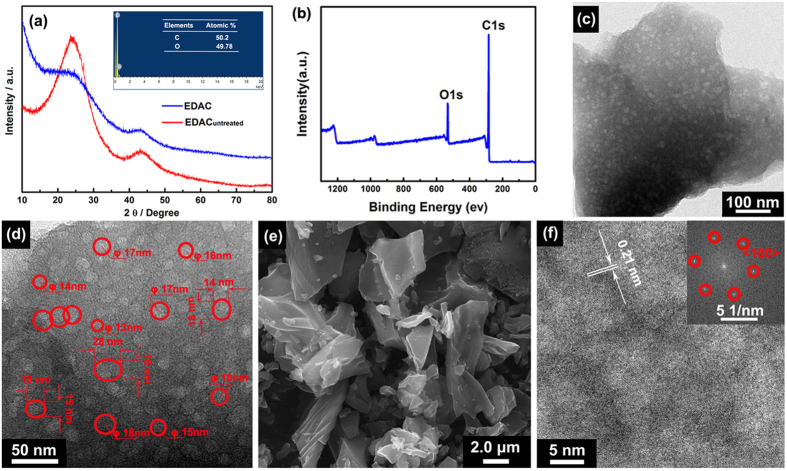
(**a**) XRD pattern of the samples for comparison and the EDS spectrum of EDAC (inset), (**b**) XPS survey spectra of EDAC, (**c**) low-magnification, (**d**) high-magnification TEM images, (**e**) FESEM image of EDAC, (**f**) HRTEM image of EDAC (and its FFT plots inset).

The XRD patterns of the graphitized EDAC carbons are shown in Fig. 4a. The characteristic peaks at around 26° and 43° corresponds to the (002) and (100) reflections of a graphite-type carbon structure, demonstrating the graphitic structures were formed as consequence of heat treatment via high-temperature polymerization method^25^,^26^. In addition, the broader Bragg reflection peaks on activated carbon with secondary carbide processing (EDAC) than without (EDAC_untreated_) reveal that the carbonization process at high temperature is conducive to the formation of amorphous carbon, corresponding to the formation of porous structure^27^. The X-ray photoelectron spectroscopy (XPS) was used to investigate the surface states of the activated carbons. As shown in Fig. 4b, the atom ratio of C and O in EDAC is about 88.04:11.96. The oxygen is mainly assigned to the oxygen-containing groups on the surface of EDAC. The FTIR analysis was performed and shown in Fig. S2a to confirm their existence. The intensive characteristic peak at 3435 cm^−1^ originated from the stretching vibration of the O-H bond^28^. The characteristic peaks around 1380, 1623, 2840 and 2917 cm^−1^ correspond to the stretching vibration of C-O bond, C-H bond, and C=C bond, respectively^29^.

The morphology and the nanostructure of EDAC were investigated by TEM, FESEM and HRTEM technologies. Figure 4c,d show the TEM images at different magnification. Numerous crossed holes with a honeycomb-like structure are observed in the obtained EDAC. Note that, the precursor (eggplant, Fig. S2b) and the intermediate material (EDAC_untreated_, Fig. S2c,d) are nonporous. The pore network in carbons should be formed during KOH activation process via the reactions in Eqs (1–5)^30^,^31^.


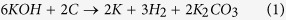



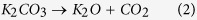



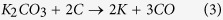



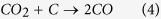



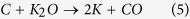


**Figure 5:**
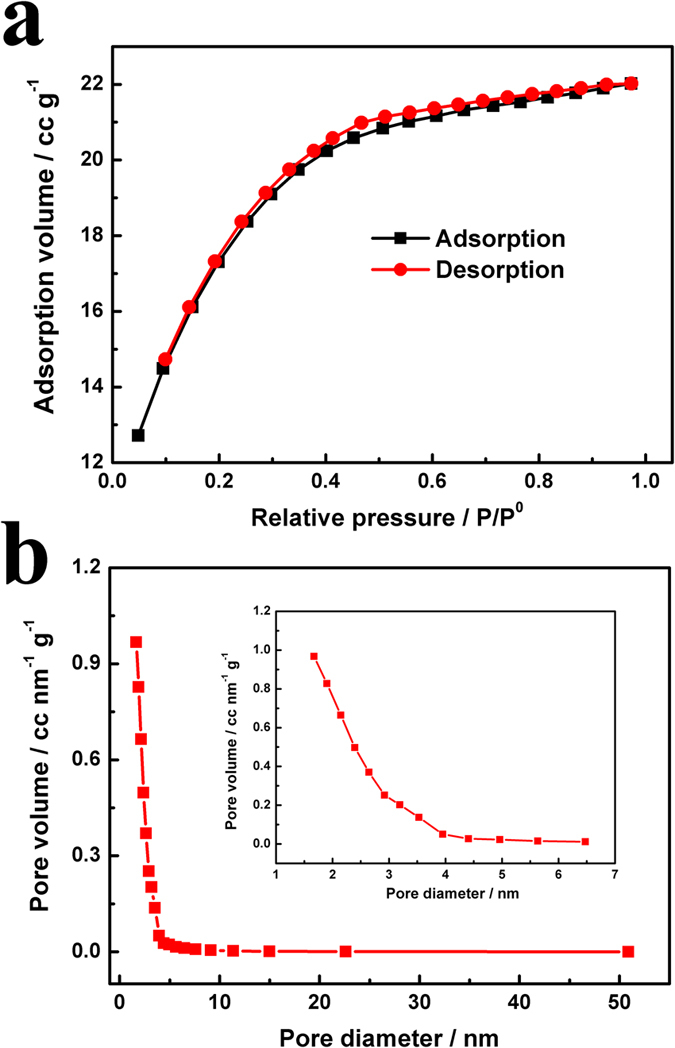
(**a**) Nitrogen adsorption/desorption isotherms and (**b**) BJH pore size distribution of EDAC carbon.

The size of holes is ranged from 0.35~70 nm, which is substantially larger than twice the size of the solvated ions (K^+^ and OH^−^), contributing capacitance from compact layers of ions residing on both adjacent hole walls^32^. The FESEM image in Fig. 4e demonstrates the uniform dispersity of the activated carbons. Figure 4f shows the HRTEM image and the FFT plots. In HRTEM image, the lattice fringe spacing was measured to be ~0.21 nm, corresponding to (100) planes of graphite-type carbon, which is consistent with the XRD result. The pore size distribution of the as-prepared activated carbon are studied by nitrogen adsorption-desorption measurement at 77 K. The I-type isotherm plots shown in Fig. 5a indicate that the microporous (≤2 nm) structure is predominant in EDAC^25^. According to t-Plot analysis, the micropore area is up to 1423.6 m^2^ g^−1^, accounting for around 51.5% of its BET surface area (2764.3 m^2^ g^−1^). Moreover, the micropore volume is as high as 0.58 cm^3^ g^−1^ (total pore volume of 1.09 cm^3^ g^−1^), which is much higher than commercial activated carbons, as shown in Table 1. Meanwhile, besides high surface area from micropores, mesopores are also observed in the EDAC (Fig. 5b). The sluggish isotherm and the high surface area derived from the pores larger than 2 nm (1340.7 m^2^ g^−1^ and 0.51 cm^3^ g^−1^ for mesopores) demonstrate the mesoporous structure of the EDAC.

**Figure 6:**
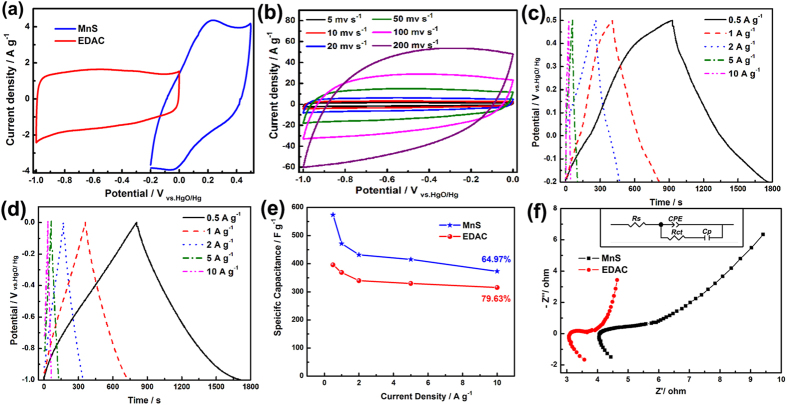
(**a**) CV curves of MnS and EDAC electrodes 5 mV s^−1^, (**b**) CV curves of EDAC electrode at different scan rates, (**c**) charge-discharge curves of MnS electrodes at different current densities, (**d**) charge-discharge curves of EDAC electrodes at different current densities, (**e**) specific capacitance calculated at different current densities, and (**f**) EIS Nyquist plots of MnS and EDAC electrodes. The insets are the corresponding equivalent circuit.

The electrochemical properties of the as-prepared MnS nanocrystals and EDAC were measured as working electrodes in a three-electrode system. As shown in Figure 6a,b, the quasi-rectangular CV curves of EDAC electrode at different scan rates demonstrate its ideal double-layer capacitive behaviour. When the scan rate reached up to 200 mV s^−1^, the remained typical rectangular shape should be ascribed to the porous structure of EDAC. The obvious redox peaks shown in CV (Fig. 6a) and charge-discharge curves (Fig. 6c) of MnS electrode can be attributed to the Faradaic redox reactions described as Eqs 6 and 7 40,41.


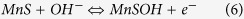



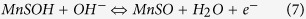


The excellent linear slope (close to equicrural triangle) from the various galvanostatic charge-discharge curves of EDAC (Fig. 6d) indicate its typical capacitive behavior^42^. The specific capacitances of the electrodes, which calculated from the charge-discharge process based on Eq. S4, are shown in Fig. 4e. Based on the laminar structure and high electronic conductivity of the MnS nanocrystal, it delivers the specific capacitance of 573.9, 471.6, 431.4, 415.0, and 372.9 F g^−1^ at 0.5, 1, 2, 5, and 10 A g^−1^, respectively. Meanwhile, benefited from the high specific surface area and high total pore volume, EDAC reveals a much high specific capacitance of 396.0, 368.6, 339.4, 329.7, and 315.3 F g^−1^ at 0.5, 1, 2, 5, and 10 A g^−1^, with a capacitance retention of 79.63%. The specific capacitance at 0.5 A g^−1^ is slightly less than the highest reported value (411 F g^−1^)^43^. The outstanding charging-discharging performance of EDAC should be ascribed to its porous nanostructure, which can not only provide a continuous electron pathway, but also facilitate short ionic transportation pathways^44^,^45^,^46^.

To further investigate the electrochemical performance of the electrodes, electrochemical impedance spectroscopy (EIS) analysis was used in the frequency range of 0.1~10^5^ Hz. The Nyquist plot of the electrodes were illustrated in Fig 6f, with a fitted equivalent circuit (inset) and the values of *R*_*s*_, *R*_*ct*_, *C*_*p*_, and *CPE* were calculated using the ZSimpWin software and the calculated values are listed in Table S1 47,48. The *R*_*s*_ is the total ohmic resistance of electrolyte resistance, intrinsic resistance of material, and contact resistance at the active material/current collector interface^49^. The *R*_*ct*_ of MnS electrode represents charge-transfer resistance, whereas the *R*_*ct*_ of EDAC is responsible for the self-discharge process. The time constant of the self-discharge is equal to *R*_*ct*_·*CPE*^47^. The low *R*_*(s+ct)*_ values of MnS (4.13 + 5.31) and EDAC (3.29 + 1.55) demonstrate their high electronic conductivity and electrochemical reactivity^50^.

Based on the excellent performances of positive and negative materials, an asymmetric supercapacitor was assembled and denoted as MnS//EDAC supercapacitor. The mass balance between electrodes was calculated on the basis of the equivalence of charges passing across the positive or the negative electrode (*q*_+_ = *q*_−_)^51^. Therefore, the mass ratio of positive and negative electrodes (m_+_/m_−_) can be calculated by Eq. 8:


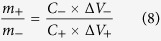


**Figure 7:**
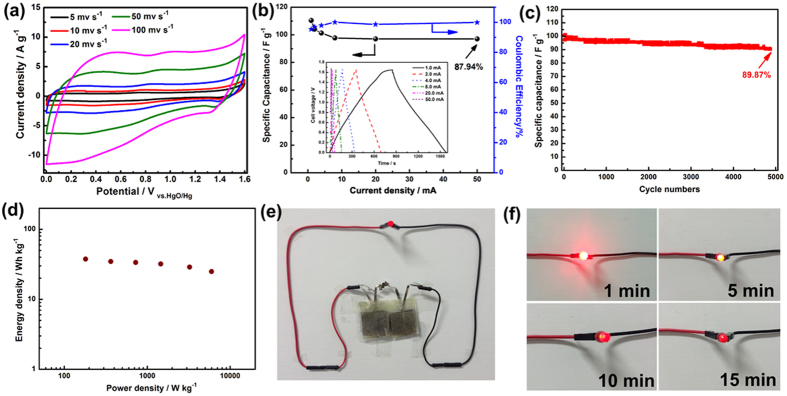
(**a**) CV curves of MnS//EDAC at different scan rates, (**b**) specific capacitance and coulombic efficiency calculated by charge-discharge curves (inset) of MnS//EDAC, (**c**) cycle performance at a current density of 1 A g^−1^, and (**d**) Ragones plots of asymmetric supercapacitors (calculated by the total active materials), (**e,f**) A red LED powered by two assembled ASC devices in series.

The loading amount of positive/negative materials in this ASCs is 2.0/2.61 mg cm^−2^, respectively. As shown in Fig. 7a, the MnS//EDAC asymmetric supercapacitor shows an ideal capacitive behavior with quasi-rectangular CV curves, even at a scan rate of 100 mV s^−1^, indicating its desirable high rate charging/discharging property for power devices. The galvanostatic charge/discharge curves (inset of Fig. 7b) show equicrural triangle shapes and are considerably symmetrical, demonstrating the good reversible performance of MnS//EDAC supercapacitor. The specific capacitance calculated by Eq. S4 and the coulombic efficiency based on Eq. S5 are shown in Fig. 7b. The calculated specific capacitances at the current densities of 1.0, 2.0, 4.0, 8.0, 20.0, and 50.0 mA are 110.4, 104.0, 101.2, 97.74, 97.09, and 97.09 F g^−1^, respectively. The high capacitance retention of 87.94% should be attributed to their higher electronic conductivity and the particular pore structure of EDAC. The lower coulombic efficiency (slightly higher than 90%) at low current density should be attributed to that the ratio of the side reactions at low current density is higher than that at high current density. The capacitance decay for the cell mainly because of the broken up of manganese sulfide nanowires, as shown in Fig. S3b~d. The original wire-like nanostructure provides transfer paths for electron, as shown in Fig. 1b. With the process of charging and discharging, the MnS nanowires broken up, which affected the electronic conduction seriously and caused the decay of capacitance. Note that, all of the coulomb efficiencies in different charge-discharge current densities are higher than 90%. In term of the cycle life, the MnS//EDAC cell shows capacitance retention of 89.87% over 5000 cycles (Fig. 7c), which should be attributed to the following respects: (1) the layered wurtzite structure of γ-MnS nanocrystal is much better accessible for the reactant molecules and cations through the interlayer space, which can be observed visually from XRD patterns at different state of charges (Fig. S3a)^52^; (2) the ideal stability of MnS nanocrystal. After 1000 charge/discharge cycles, the crystal structure is reserved even though the manganese sulfide nanowires broken up into nanoparticles (Fig. S3b~d); (3) the particular pore structure of EDAC. When holes were substantially larger than twice the size of the solvated ions (OH^−^), there was a contribution to capacitance from compact layers of ions residing on both adjacent hole walls^31^. Ragone plots, depicting the relationship of power densities (P) and energy densities (E), which were calculated by Eqs S6 and S7 are shown in Fig. 7d. The energy density reaches up to 37.6 Wh kg^−1^ at a power density of 181.2 W kg^−1^. Even at the high power density of 5976 W kg^−1^, the energy density remains as high as 24.9 Wh kg^−1^. The energy densities are much higher than other asymmetric supercapacitors in aqueous electrolyte, such as AC//AC (<10 Wh kg^−1^)^53^, CNT//CNT (<10 Wh kg^−1^)^54^, FeOOH//MnO_2_ (24 Wh kg^−1^)^55^, activated carbon//MnO_2_ (17.3 Wh kg^−1^)^56^. Furthermore, we assembled two ASCs in series (the loading amount of MnS/EDAC are 5.74/5.81 and 5.96/5.89 mg cm^−2^, respectively) with saturated potassium hydroxide agar gel as separator and electrolyte and lighted up a red LED indicator for 15 minutes, as shown in Fig. 7e,f. All these results demonstrated their practical application merit intuitively.

## Conclusions

In summary, a low-cost high-performance asymmetric supercapacitors (ASCs) has been assembled using γ-MnS as positive electrode and EDAC as negative electrode with saturated potassium hydroxide agar gel as the separator and electrolyte. Because of the special nanostructure of MnS and EDAC, the MnS//EDAC asymmetric supercapacitor shows high specific capacitance, outstanding energy density, excellent cycling stability at an operating voltage of about 1.60 V. Impressively, such two assembled all-solid-state cells in series can light up a red round LED indicator for 15 minutes after fully charged. The inexpensive and pollution-free materials and the remarkable electrochemical performances will enable them a good practical application prospect.

## Additional Information

**How to cite this article**: Chen, T. *et al.* All-solid-state high performance asymmetric supercapacitors based on novel MnS nanocrystal and activated carbon materials. *Sci. Rep.* **6**, 23289; doi: 10.1038/srep23289 (2016).

## Supplementary Material

Supplementary Information

## Figures and Tables

**Table 1:** BET specific surface area, micropore Area, micropore volume, average pore diameter and specific capacitance at 0.5 A g^−1^ of EDAC and other commercial activated carbons^33^,^34^,^35^,^36^,^37^,^38^,^39^.

Sample	BET SSA (m^2^ g^−1^)	Micropore Area (m^2^ g^−1^)	Micropore volume (cm^3^ g^−1^)	Average pore diameter (nm)	Specific capacitance (F g^−1^)
EDAC	2764.3	1423.6	0.58	1.7	396.0
Black Pearl 2000	1379	934	0.22	8.6	100.7
Ketjen Black EC 600J	1439	189	0.14	6.5	45.3
Vulcan XC-72	213	114	0.06	9.4	15.2
Acetylene Black	1304	184	0.0045	3.1	4.1
