# Supplementary material for: All-solid-state high performance asymmetric supercapacitors based on novel MnS nanocrystal and activated carbon materials
Source: Sci Rep. 2016 Mar 29;6:23289. doi: 10.1038/srep23289 (PMC4810367; doi:10.1038/srep23289)
Supplement: Supplementary Information [file srep23289-s1.doc]

**All-solid-state high performance asymmetric supercapacitors based on novel MnS nanocrystal and activated carbon materials**

Teng Chen,^[a]^ Yongfu Tang,[[1]](#footnote-2)*[a] Yuqing Qiao,^[a]^ Zhangyu Liu,^[a]^ Wenfeng Guo,^[a]^ Jianzheng Song,^[a]^ Shichun Mu,^[b]^ Shengxue Yu,^[a]^ Yufeng Zhao,^[a]^ Faming Gao^[a]^

^[a]^ Key Laboratory of Applied Chemistry, College of Environmental and Chemical Engineering, Yanshan University, Qinhuangdao, 066004, China

^[b]^ State Key Laboratory of Advanced Technology for Materials Synthesis and Processing, Wuhan University of Technology, Wuhan, 430070, China

**Experimental Section**

**Preparation of MnS nanocrystal.**

The MnS nanocrystal was prepared via a facile template-free hydrothermal method. Specifically, 0.1 g MnCl_2_·4H_2_O was dissolved in 50 ml distilled water under vigorous stirring. Then, 0.2 g thiourea, which has dissolved in 10 ml 0.02 M KOH, was added dropwise. Subsequently, the mixture was transferred into a 100mL Teflon-lined stainless-steel autoclave and heated at 120 ℃ for different times (20 min, 2 h, 5 h, and 8 h). The powders were obtained after being washed, centrifuged and dried.

**Preparation of eggplant derived activated carbon (EDAC).**

Eggplant was peeled with a vegetable peeler, crushed, and dried at 80℃for 12 h. After pulverization in a mortar, the powder-like eggplant was heated to 300℃with a heating rate of 1℃min^-1^ and sustained at that temperature for 3 h at Ar atmosphere. The product, which was denoted as EDAC_untreated_, was obtained after washing and drying. The as-prepared product was activated with KOH and HNO_3_ following the procedure as : the as-prepared product and KOH were mixed at a weight ratio of 1:2 (W_EDAC untreated_/W_KOH_), and then heated to 700℃at Ar atmosphere with a heating rate of 10℃min^-1^ and sustained at that temperature for 4 h through a vapor deposition polymerization technique. Subsequently, the samples was sonicated in 2 M HNO_3_ for several times and then cleaned with distilled water for several times. The sample was obtained after washing and drying and denoted as EDAC.

**Physicochemical characterizations.**

The morphology and microstructure of the as-prepared samples were characterized using transmission electron microscopy (TEM, HT7700), high-resolution transmission electron microscopy (HRTEM), and filed emission scanning electron microscopy (FESEM, KYKY-2800B). The compositions and phase structure were investigated by X-ray diffraction (XRD) spectra (Bruker AXS D8 diffractometer with Cu K Rradiation). Fourier transform infrared (FTIR) measurements of the samples were performed on Nicolet 380 FT-IR using KBr method.

**Fabrication of electrode, solid electrolyte and electrochemical measurements.**

The positive working electrode was prepared by mixing MnS nanocrystal, acetylene black and polytetrafluoroethylene (PTFE) at a weight ratio of 70:15:15, meanwhile, the negative electrode was prepared by mixing EDAC and polytetrafluoroethylene (PTFE) at a weight ratio of 9:1. After ultrasonic dispersing for 20 min, the mixture was coated onto Ni foam and dried in vacuum at 80 ^o^C for 12 h. The electrochemical tests including cyclic voltammetry (CV) and electrochemical impedance spectroscopy (EIS, 0.05 Hz-100 KHz) were carried out via a CHI 660A workstation, galvanostatic charge-discharge (GC) was performed on Land CT 2001A. All the measurements were carried out in 2.0 M KOH electrolyte with a conventional three-electrode system where Hg/HgO electrode and Pt foil (1×1 cm^2^) were used as reference electrode and counter electrode, respectively.

In asymmetric supercapacitors, we used saturated potassium hydroxide agar gel as separator and electrolyte. The detailed preparation process is followed: 1.0 g of agar gel placed in culture dish and reasonable amount of saturated potassium hydroxide solution added slowly with continuous stirring for 20 minutes. The mixture was heated at 70 ℃ until converted to jelly. The asymmetric supercapacitors were assembled by employing MnS nanocrystal and eggplant derived activated carbon (EDAC) as positive and negative materials, using the jellylike solid electrolyte as separator and electrolyte. The specific fabrication process was described as follow: put the as-prepared electrode slices in 2 M KOH solution for 2 h, making the active materials complete wetting. Then, cut the solid electrolyte into electrode shape with a thickness of about 2 mm. Put the electrodes and solid electrolyte in a pouch in the order of MnS//electrolyte//EDAC. A pouch cell was assembled after sealing the pouch. All the electrochemical measurements were based on the cell of MnS/EDAC (2.0/2.61 mg cm^-2^). In order to light up a red LED indicator for a longer time, we assembled two ASCs in series (the loading amount of MnS/EDAC were 5.74/5.81 and 5.96/5.89 mg cm^-2^, respectively).

**Supplementary Equations**

$NH_{2}SNH_{2}+OH^{-}\to HN=NH+SH^{-}+H_{2}O$ S1

$SH^{-}+OH^{-}\to S^{2-}+H_{2}O$ S2

S3

$C_{s}=\frac{I\cdot\Delta t}{\Delta V\cdot m}$ S4

where *C_s_* (F g^-1^), *I* (A), *∆*t (s), *∆V* (V) and m (g) represent the specific capacitance, discharge current, discharge time, potential range of discharge and the mass of active material.

$CE=\frac{C_{sd}}{C_{sc}}\times100\%$ S5

where C_sc_ and C_sd_ were the specific capacitance of charge and discharge

process, respectively.

$E=CV^{2}/2$ S6

where E, C and V are the specific energy density (Wh kg^-1^), cell capacitance (F g^-1^), and operating potential window(V), respectively.

$P=E/\Delta t$ S7

where E, P and △t are the specific energy density (Wh kg^-1^), power density (kW kg^-1^), and discharge times(s), respectively.

**Supplementary Tables**

**Table S1. Potential changes and mass specific capacitance in each electrode at three electrode system with different mass loadings of the active material at 0.5 A g^-1^.**

| Active material with different mass loadings  / mg cm^-2^ | MnS  (2.0) | MnS  (5.74) | MnS  (5.96) | EDAC  (2.61) | EDAC  (5.81) | EDAC  (5.89) |
| --- | --- | --- | --- | --- | --- | --- |
| Potential changes / V | -0.2~0.5 | -0.2~0.5 | -0.2~0.5 | -1~0 | -1~0 | -1~0 |
| Specific capacitance / F g^-1^ | 573.9 | 558.4 | 554.7 | 396.0 | 390.4 | 390.2 |

**Table S2.** Calculated values of *Rs*, *Rct, CPE, and Cp* of the obtained supercapacitor electrodes through fitting of the experimental impedance spectra based on the proposed circuit.

| **Samples Rs (Ω·cm^-2^) Rct （Ω·cm^-2^） CPE(mF) Cp（F）** |
| --- |
| MnS 4.13 5.31 0.43 0.31 |
| EDAC 3.29 1.55 0.59 0.14 |

Where ***Rs*** is the combined resistance of electrolyte resistance, intrinsic resistance of material, and contact resistance at the active material/current collector interface, ***R_ct_*** of MnS is related to the faradaic charge-transfer resistance, ***R_ct_*** of EDAC responsible for the self-discharge and the time constant of the self-discharge is equal to ***R_ct_***∙***CPE***, ***CPE*** is electric double-layer capacitor and ***C_p_*** is the faradaic pseudocapacitor.

**Supplementary Figures**

**Figure. S1.** TEM images of MnS nanocrystals obtained from the hydrothermal coprecipitation process at 20 mins (a), 2 h (b), 5 h (c), and 8 h (d).


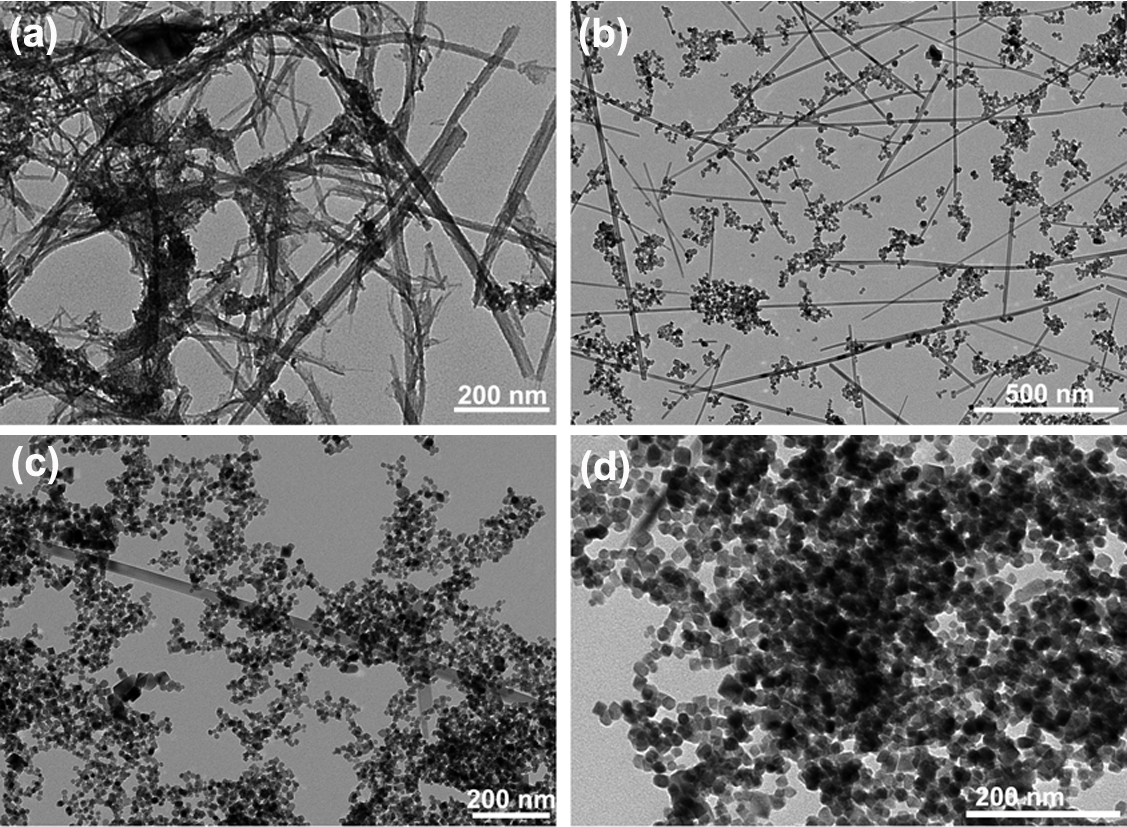


**Figure.S2.** (a) FTIR spectra of the obtained samples, (b) TEM image of eggplant, (c) TEM image, and (d) FESEM image of intermediate material (EDAC_untreated_).


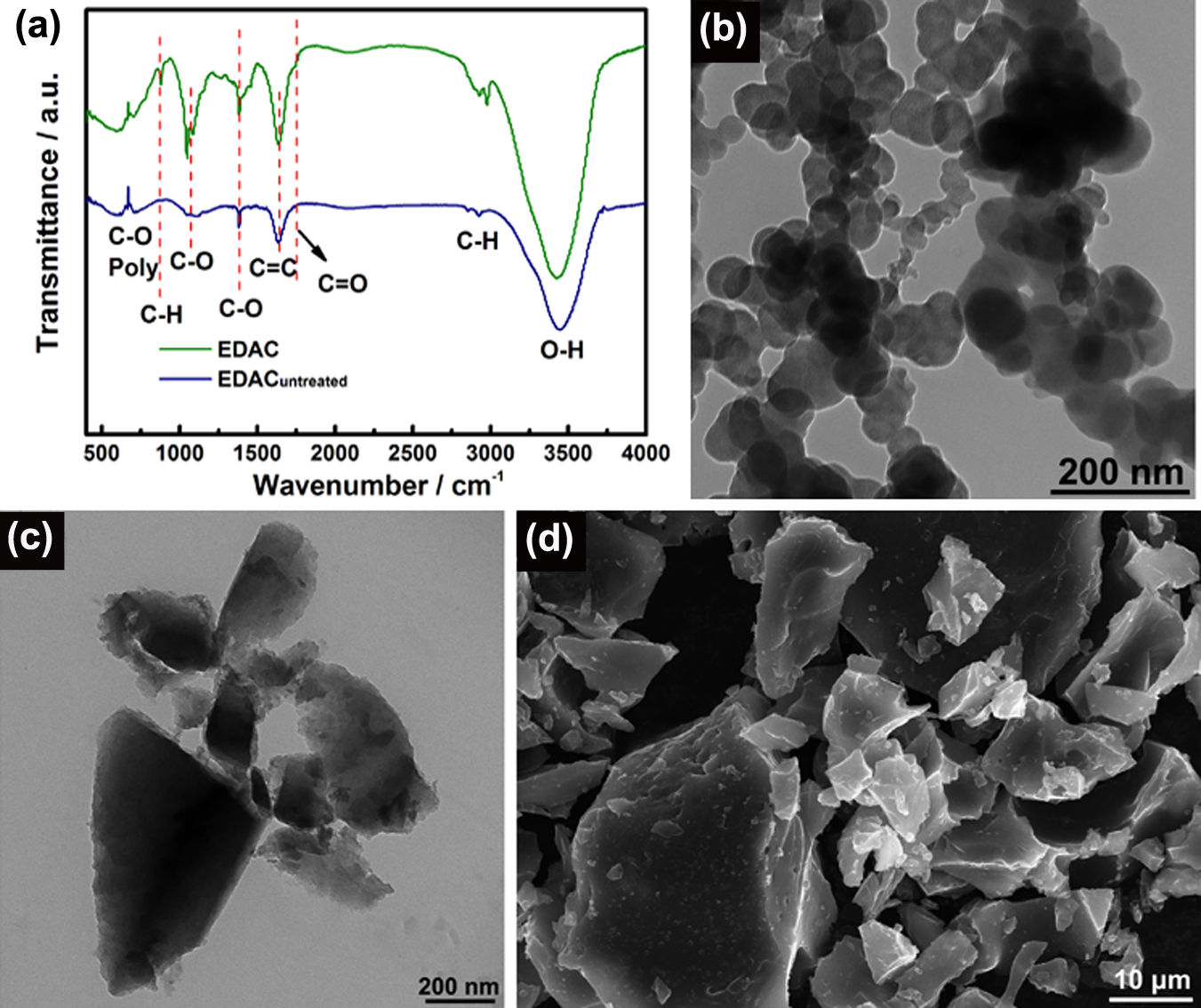


**Figure. S3.** (a) XRD patterns of MnS electrode at different state of charges (SOC), (b) SEM image, (c) low magnification, and (d) high magnification TEM images of MnS nanocrystals after 1000 charge/discharge cycles.


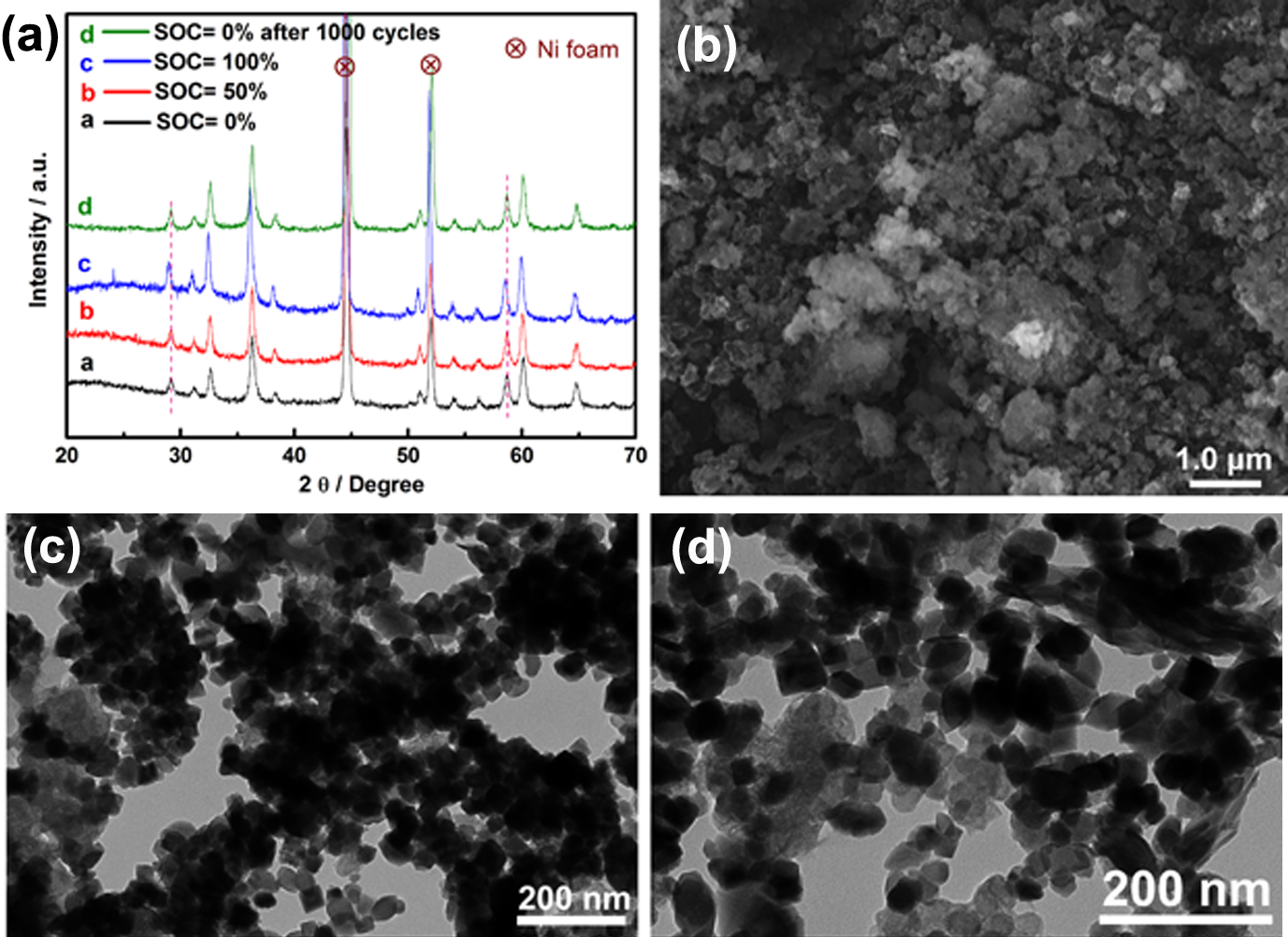


1. ^*^ Corresponding author:

   E-mail address: [tangyongfu@ysu.edu.cn](mailto:tangyongfu@ysu.edu.cn) (Y. Tang) [↑](#footnote-ref-2)
